# A Method of Extracting Ontology Module Using Concept Relations for Sharing Knowledge in Mobile Cloud Computing Environment

**DOI:** 10.1155/2014/382797

**Published:** 2014-08-27

**Authors:** Keonsoo Lee, Seungmin Rho, Seok-Won Lee

**Affiliations:** ^1^Graduation School of Information & Communication, Ajou University, Suwon, Republic of Korea; ^2^Department of Multimedia, Sungkyul University, Anyang, Republic of Korea; ^3^Department of Software Convergence Technology, Ajou University, Suwon, Republic of Korea

## Abstract

In mobile cloud computing environment, the cooperation of distributed computing objects is one of the most important requirements for providing successful cloud services. To satisfy this requirement, all the members, who are employed in the cooperation group, need to share the knowledge for mutual understanding. Even if ontology can be the right tool for this goal, there are several issues to make a right ontology. As the cost and complexity of managing knowledge increase according to the scale of the knowledge, reducing the size of ontology is one of the critical issues. In this paper, we propose a method of extracting ontology module to increase the utility of knowledge. For the given signature, this method extracts the ontology module, which is semantically self-contained to fulfill the needs of the service, by considering the syntactic structure and semantic relation of concepts. By employing this module, instead of the original ontology, the cooperation of computing objects can be performed with less computing load and complexity. In particular, when multiple external ontologies need to be combined for more complex services, this method can be used to optimize the size of shared knowledge.

## 1. Introduction

Mobile cloud computing environment consists of two concepts [1]. One is the mobile computing and the other is cloud computing. The mobility in computing environment means that users can access any computing service in spite of the spatial limitation. With the popularization of mobile devices, such as smart phone and tablet, the spatial limitation in computing environment seems to be resolved. However, the inferior computing power and low battery capacity of mobile devices make it difficult to realize the mobility in computing environment. Cloud computing is what can disengage these demerits of mobile devices. In cloud computing environment, complex services can be provided to the users who own low-spec systems such as mobile devices. In order to make this magical performance, cooperation and collaboration of various distributed computing objects in the cloud should be guaranteed.

Knowledge sharing among the computing objects is one of the most efficient methods to assure the cooperation and the collaboration in this mobile cloud computing environment [2, 3]. Even if the knowledge is hard to make and expensive to manage, intelligent behaviors can be made easily using knowledge in dynamically changing environment. By sharing knowledge, more complex services can be executed with less computing power and with higher accuracy. Ontology has been recommended as a knowledge representation method since the new web environment, which is known as semantic web, was proposed around 2001 [4, 5]. Various languages, such as RDF, DAML, OIL, and F-logic, have been proposed to make ontology. In 2004, W3C introduced OWL as the official language for ontology [6]. Thereafter, various types of knowledge bases are created in ontology and opened to the public [7].

Ontology provides an easy way of using knowledge by reducing the cost of generating and managing the knowledge. And at the same time, the side effect of this convenience had occurred. Each computing object in the cloud has made its own knowledge. Ironically, the easiness of managing knowledge makes it more difficult to share the knowledge. As the team, which consists of star players but is not properly organized, cannot win the team that consists of the unnamed but is well organized, knowledge sharing should be the first and then the quantity of the knowledge. The key of resolving this problem is that all the knowledge of every computing object in the cloud does not have to be shared. The knowledge, which is necessary in cooperation, is a subset of the whole knowledge. With this subset of knowledge, which is called the ontology module, the cooperation can be made [8]. In this paper, we propose a new method of extracting ontology module based on the concept relations. By using module instead of original ontology, the cost of managing knowledge can be significantly reduced. In order to execute as a proper ontology module, it should shrink in size but not in semantics.

## 2. Background

### 2.1. Terminologies for Modularity

The concept of module, used in this paper, can be characterized as follows [8, 9]. Firstly,* knowledge module* is defined as a subpart of the entire knowledge. Secondly, the combination of modules is also a module. Thirdly, the semantics of module should not be changed even if the module is extended with other modules. And the last is that the identity of module is defined not by the contents of modules but by the functionality. In order to compare the functionality, we need a “signature” as the input parameter of the functionality. The* signature* is a set of concepts which are used to make queries which are sent to the knowledge module. Let us assume that there are two modules:* M1* and* M2*. The union of* M1* and* M2* is also a module of* M3*. When a signature, which consists of concepts* C1*,* C2*, and* C3*, is given, if the answers for the queries, which are made by the signature, of* M1* and* M3* are the same, we can say that* M1* and* M3* have the same functionality for the given signature. In this example,* M2* should not be semantically dependent on* M1* because every module should be self-contained. In this case,* M3* is called the* conservative extension* of* M1* because the semantics of* M1* is conserved in* M3* even if* M2* is added. As* M1* and* M3* have the same functionalities for the given signature, it will not matter whether to use* M1* or* M3*. But, to make efficiency in knowledge management,* M1* must be the right choice.

Therefore, the right module for the given signature will be the module which has the smallest size among the modules which have the same functionality.

### 2.2. Ontology as Knowledge Representation

In semantic web, ontology is used for representing knowledge. Generally, ontology is defined as a formal and explicit specification of a commonly shared conceptualization [3]. This ontology can be represented by various languages such as RDF, DAML, OIL, or F-logic. Ontology represents knowledge as concept and role. Concept is the term of indicating idea in a given domain. And role is the relations among concepts. OWL is an official ontology language proposed by W3C [6]. OWL has many sublanguages such as OWL-Full, OWL-DL, and OWL-Lite. In 2009, OWL version 2 was proposed and OWL2-EL, OWL2-QL, and OWL2-RL are included in this version. These classifications are based on the expressive power shown in description logic. Description logic is a family of formal knowledge representation languages and it is more expressive than propositional logic but less expressive than first-order predicate logic [9]. OWL2-EL has expressive power of ELRO, which includes concept intersection, existential restriction, and nominal and role inclusion axioms. And OWL2 has expressiveness of SROIQ; OWL-DL has expressiveness of SHOIN; and OWL-Lite has expressiveness of SHIF.

With higher expressive power, the reasoning cost becomes more expensive. Even with OWL-DL's expressive power, the reasoning task can be undecidable. Therefore, we need to choose the proper position between expressiveness and reasoning complexity [10–12]. The three major bioinformatics terminologies such as Snomed [13], Galen [14], and GO [15] are represented in EL. Therefore, we use EL as basic expressiveness of ontology. The expressive power of EL includes concept constructors, such as top (*⊤*), conjunction (*C*∩*D*), and existential restriction (∃*r* · *C*), and atomic role constructors. For this weak expression power, complex knowledge may not be expressed in EL but the knowledge can guarantee the decidability in reasoning [16].

## 3. Ontology Module Extraction

**Figure 1:**
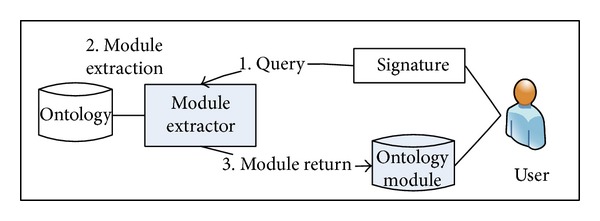
The proposed model.

The goal of the proposed method is to extract module from the original ontology according to the given signature. As shown in Figure 1, the module extractor (*ME*), which is the implementation of the proposed method, receives the signature and, according to the signature, extracts module from the original ontology. The module extraction process follows these steps. In the first place, the signature is given. Then* ME* finds the concepts, which are related to the given signature, by comparing the syntactic structure of concepts. These selected concepts become the candidates who can be the module's elements. Then, the semantic comparing process filters the unnecessary concepts for the module and removes them from the candidates' pool. Then, the remaining concepts are returned as ontology module for the given signature.

**Figure 2:**
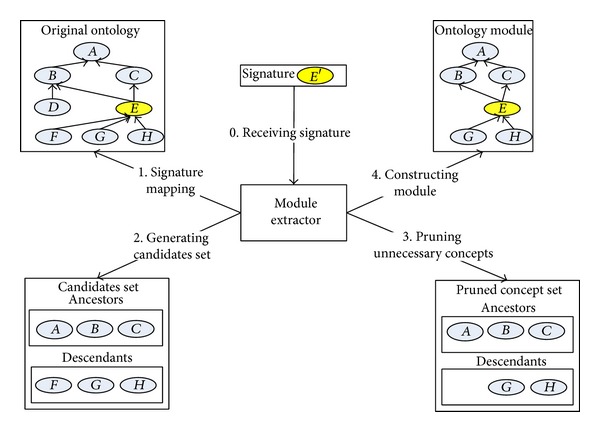
Process of module extractor.

The process of* ME* consists of two steps. One is syntactic structure mapping. The other is semantic filtering. The first syntactic part is executed by the following steps. When a signature and original ontology are given,* ME* compares the signature with concepts in the original ontology. This process can be regarded as concept mapping. According to the structural comparisons, the equivalent concept in original ontology can be extracted. As we assume that the ontology is expressed in EL, structural subsumption algorithm can be used [17]. When the equivalent concept in the original ontology is found, its descendant concepts are classified into the candidates' set. And the equivalent concept's ancestor concepts are also classified into the set. In Figure 2, an example of this proposed method is shown. When the signature *E*′ is provided, it is mapped into the original ontology's concept *E*. Then, as a syntactic part, concepts* A*,* B*, and* C,* which are concept* E*'s ancestor concepts, and concepts* F*,* G*, and* H*, which are concept* E*'s descendant concepts, become the candidates for the ontology module. The candidate concepts are selected according to the syntactic relation with the equivalent concept which is mapped to the signature. In ancestors' list, concept* E*'s direct ancestors* A*,* B*, and *C* are inserted. In descendants' list, all the subsumed concepts by concept *E* are inserted. And the ancestors of concepts in descendants' list are also inserted into the ancestors' list. As this process finds all the concepts which have direct or indirect relations with the equivalent concept, the size of candidates' set is almost the same as that of the original ontology's size. In order to reduce the size of the module, the unnecessary concepts need to be removed. The criteria, which distinguish concepts, which are related to the signature, from the unrelated concepts, are the the most important parts in the proposed method.

After the syntactic process is over, the semantic filtering is executed. This process is also to reduce the size of the candidates' set. In semantic level, the criteria for deciding the relation of concept to the signature consist of three rules. The first rule is that the removed concept should not affect the relations among the remaining concepts. This rule is to preserve the conservative extension constraint of the module. For example, when three concepts, which relate to each other as *A*⊆*B* and *B*⊆*C*, exist, concept *B* should not be removed. Without *B*, the relation between *A* and *C* cannot be inferred. The second rule is to prune the unnecessary descendant concepts. All the concepts in the candidates' set are related to the signature with subsumption relation. However, some concept, which is subsumed by the signature, may be identified by other concepts. Such concept can be removed from the signature's module. For example, “doctor” concept has three child concepts such as “female doctor,” “male doctor,” and “ophthalmologist.” Two of them are subsumed by “gender” concept and one is subsumed by “specialty.” For the descendants, the concept, which is too specific, can be removed too. The specification of concept is relative. Therefore, the number of siblings and the depth of concept in the ontology hierarchy are used to evaluate the concept's specification level. For example, the single concept, which locates in the leaf position in the hierarchy, can be removed. And the concept “male doctor with age of 31” is also classified as too-specific-concept if there is no other concept which is specified with age. Even if there are other concepts with “age,” it can be regarded as too-specific-concept when the ranges of “age” are different from each other. From this difference, the intensity of concept relation can be inferred. Through this relation weight, the descendant concepts, which have weaker relation than the predefined threshold value, are removed from the candidates set. The last rule is to remove the unnecessary ancestor concepts. In order to apply this rule, the second rule needs to be executed in advance. By considering the remaining descendant concepts, unnecessary ancestor concepts can be pruned. From the second rule, “female doctor” and “male doctor” concepts remained. These concepts have weak relations to the “specialty” concept, which subsumes the “doctor” concept. Therefore, this “specialty” concept can be pruned from the candidates set. As shown in Figure 2, the 3rd step prunes the unnecessary concepts: *F* in descendant concepts and *B* in ancestor concepts. Then the remaining concepts are reconstructed in the 4th step.

After the* ME*'s execution, ontology module for the given signature is made from the original ontology. As the threshold value, used in this example, is ontology sensitive, it needs to be modified according to the original ontology's structure and each given signature. The retrieved ontology module's appropriateness can be verified by comparing the reasoning result of module and the original ontology. If the same result is returned by both ontologies, the extracted module can be considered as success. The algorithm of* ME* is shown in Algorithms 1, 2, 3, and 4. In Algorithms 1, 2, 3, and 4, several functions are employed without detailed procedures. The roles of these functions are as follows. The function “*Subsumption_Check(Concpet A, Concept B)*” checks the subsumption relation between the concepts in parameters. If* A* subsumes* B*, it returns true value. The functions, which are in “*Push_XXXList(Concept)*” form, push the parameter into the specified list. The “*Pop_XXXList(Concept)*” does the opposite action. The “*inXXXList(Concept)*” function checks whether the concept is in the list or not. The “*IncreaseCount(Concept)*” function counts the number of concept's selections. And “*Count(Concept)*” returns the number. The “*numOfsubsume(Concept)*” function counts the number of the given concept's descendants. “*Ancestor(Concept), *” “*Descendants(Concept), *” and “*Parents(Concept)*” return the list of the given concept's ancestors, descendants, and parents, respectively. The “*Generate_Hierarchy(List)*” makes hierarchy of the given lists by calculating the subsumption relations among the list's elements. The variable threshold is set according to the employed domain ontology.

## 4. Conclusion

In mobile cloud computing environment, the cooperation of computing objects in cloud is one of the most important features to make a complex service to users with mobile device. To make this cooperation work properly, the computing objects need to share their knowledge. Ontology modularization can reduce the cost of sharing knowledge. But this advantage can be provided only if the semantics of the module is the same as the original ontology. For this goal, this paper proposes a new way of extracting module from the original ontology by distinguishing between related concepts and unrelated concepts for the given signatures. Through this classification, only the concepts that are related to the given signature can be extracted and the set of all the selected concepts becomes the proper module for the signature, which is smaller in size but has the same semantics compared to the original ontology. By sharing this module, the cooperation among computing objects can be executed with more intelligence and less expense.

## Figures and Tables

**Algorithm 1:**
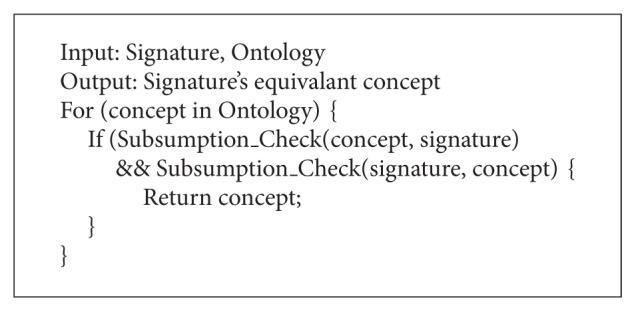
Algorithm of module extraction (signature mapping step).

**Algorithm 2:**
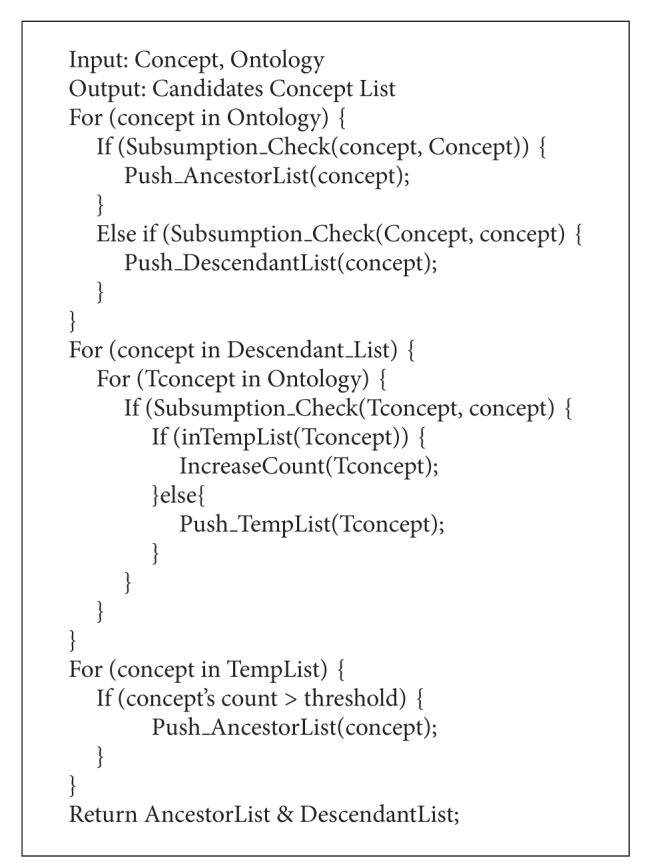
Algorithm of module extraction (candidates concept list extracting step).

**Algorithm 3:**
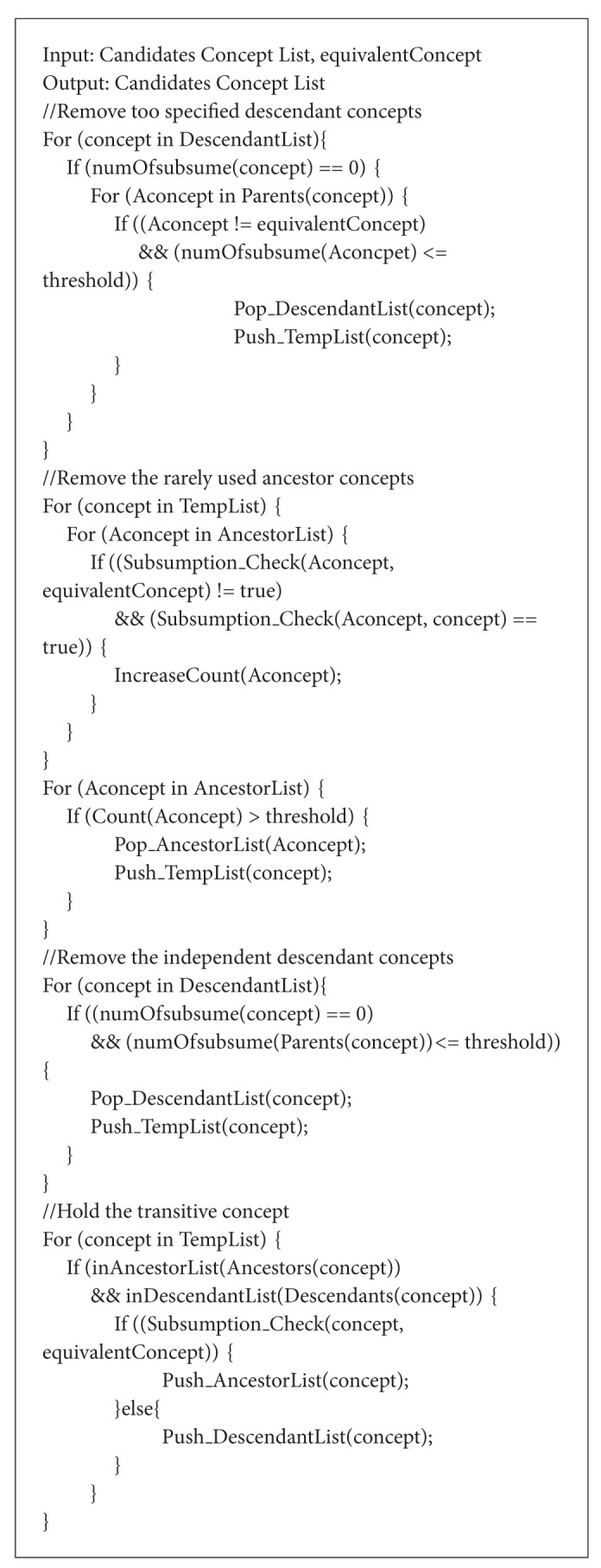
Algorithm of module extraction (concept pruning step).

**Algorithm 4:**
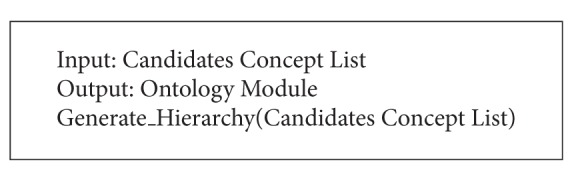
Algorithm of module extraction (constructing module hierarchy step).
